# Does the painDETECT questionnaire identify impaired conditioned pain modulation in people with musculoskeletal pain? – a diagnostic accuracy study

**DOI:** 10.1186/s40945-023-00171-8

**Published:** 2023-09-18

**Authors:** Juliana Valentim Bittencourt, Eduardo Gallas Leivas, Arthur de Sá Ferreira, Leandro Alberto Calazans Nogueira

**Affiliations:** 1 Rehabilitation Science Postgraduate Program at Augusto Motta University Centre (UNISUAM), Avenida Paris, 84, Bonsucesso, CEP, Rio de Janeiro, 21041-020 RJ Brasil; 2 Physiotherapy Department at Federal Institute of Rio de Janeiro (IFRJ), Rio de Janeiro, Brazil

**Keywords:** Musculoskeletal pain, Neuropathic pain, Pain mechanisms, Central nervous system sensitization, Diffuse noxious inhibitory control

## Abstract

**Background:**

People with neuropathic-like symptoms had more unfavourable pain features than people with nociceptive. Moreover, deficient conditioned pain modulation is common in people with neuropathic-like symptoms. PainDETECT questionnaire have been used to assess the central sensitisation sign and symptoms. However, whether the painDETECT questionnaire can identify the conditioned pain modulation's impairment is still unknown. Therefore, the current study aimed to evaluate the diagnostic accuracy of the painDETECT questionnaire in detecting the impairment of conditioned pain modulation in people with musculoskeletal pain.

**Methods:**

We conducted a diagnostic accuracy comparing the painDETECT questionnaire (index method) with the cold pressor test, the psychophysical test used to assess the conditioned pain modulation (reference standard). We determined diagnostic accuracy by calculating sensitivity, specificity, predictive values, and likely hood ratios.

**Results:**

We retrospectively enrolled 308 people with musculoskeletal pain in outpatient departments. Most participants were female (n 20 = 220, 71.4%) and had a mean age of 52.2 (± 15.0) years. One hundred seventy-three (56.1%) participants were classified as nociceptive pain, 69 (22.4%) as unclear, and 66 (21.4%) as neuropathic-like symptoms. According to the cold pressor test, 60 (19.4%) participants presented impairment of conditioned pain modulation. The cutoff point of 12 of the painDETECT questionnaire showed values of diagnostic accuracy below 70% compared to the cold pressor test, except for a negative predictive value [76.9 95% Confidence Interval (CI) 71.7 to 81.5]. The cutoff point 19 showed high specificity (78.6%, 95% CI 73.0 to 83.5), high negative predictive value (80.5%, 95% CI 78.1 to 82.7), and accuracy of 67.5% compared to the cold pressor test.

**Conclusion:**

The painDETECT questionnaire seems valuable for ruling out people with musculoskeletal pain and impairment of conditioned pain modulation.

**Supplementary Information:**

The online version contains supplementary material available at 10.1186/s40945-023-00171-8.



## Background

Neuropathic pain leads to unfavourable outcomes and remains a major clinical challenge. People with neuropathic-like symptoms showed unfavourable pain features (i.e., pain intensity and functional limitation) compared to their counterparts [1]. Previous studies showed that neuropathic pain interferes with several aspects of an individual’s life, such as poor sleep quality [2], physical impairment [3], and a large psychosocial burden [4]. Moreover, there is a high prevalence of depression among people with neuropathic pain which harms the quality of life [5]. There are screening tools capable of identifying key neuropathic-like symptoms. PainDETECT questionnaire is a reliable, simple, and validated screening tool for identifying neuropathic-like symptoms in people with chronic low back pain [6]. PainDETECT questionnaire has been validated for various illnesses, including rheumatoid arthritis, osteoarthritis, fibromyalgia, cancer pain and lumbar spondylolisthesis [7]. Furthermore, compared to other available instruments, the painDETECT questionnaire is one of the best options for screening neuropathic-like symptoms (presenting 85% of sensitivity, 80% of specificity, and 83% of positive predictive accuracy) [8]. Thus, using the painDETECT questionnaire is popular among researchers and clinicians to identify neuropathic-like symptoms in people with musculoskeletal pain.

Neuropathic pain is involved with peripheral and central sensitisation (CS). Several instruments are available to identify the clinical features of CS in the musculoskeletal population [9]. The cold pressor test is one of the most appropriate conditioned pain modulation paradigms to assess descending nociceptive modulatory pathways [10]. People with neuropathic-like symptoms have impairment of pain modulation, which is considered indicative of CS-related signs and symptoms [11, 12]. For instance, carpal tunnel syndrome [13], painful diabetic neuropathy [14], painful peripheral neuropathy [15], and complex-regional pain syndrome [16] have a deficient conditioned pain modulation. Likewise, people with presumably nociceptive pain have demonstrated CS-related signs and symptoms. Lluch et al. showed that 28 to 34% of people with osteoarthritis knee pain had CS-related signs and symptoms considering different aspects of CS-related signs and symptoms (i.e., clinical manifestations of CS, quantitative sensory testing results, dysfunctional endogenous nociceptive inhibition, and neuroimaging) [17]. Additionally, the interaction between osteoarthritis knee and CS increased nocturnal discomfort and disability [18]. Physiotherapy approaches (e.g. education, exercise, manual therapy and transcutaneous electrical nerve stimulation) can target particular pain phenotypes and individualise care [19]. Therefore, detecting the impairment of conditioned pain modulation in people presenting musculoskeletal pain (i.e., nociceptive pain and neuropathic-like symptoms) may assist clinicians in offering appropriate therapeutic strategies for these groups.

The use of the painDETECT questionnaire in assessing CS-related signs and symptoms has been advocated. PainDETECT was designed as a screening tool, but it may also function as a measure of characteristics that point to enhanced central pain processing [20]. Gwylim et al. revealed that people with higher painDETECT questionnaire scores had more CS signs [21]. Likewise, the modified painDETECT questionnaire may assist identification of CS in adults with knee osteoarthritis since higher modified painDETECT questionnaire scores (> 12) were 5.6 times more likely to have CS-related signs and symptoms [22]. Of note, the prior study screened people with knee osteoarthritis using the modified painDETECT, which targets symptoms ‘in or around’ each knee rather than their “main area of pain”, pain spreading up or down from the knee, and a figure with gender-neutral [22]. It is unclear whether the painDETECT questionnaire results may detect people with impairment of pain modulation. Therefore, the current study aimed to evaluate the diagnostic accuracy of the painDETECT questionnaire in detecting the impairment of conditioned pain modulation in people with musculoskeletal pain.

## Methods

### Study design and ethical considerations

We conducted and reported a diagnostic accuracy study following the Standards for Reporting of Diagnostic Accuracy Studies (STARD) guidelines [23] (Additional file 1). The Research Ethics Committee of the Federal Institute of Rio de Janeiro approved this study (number: 02228818.0.3001.5258) following the Helsinki Declaration for research in humans. All people with musculoskeletal pain who met the eligibility criteria signed the informed consent form before undergoing the study procedures.

### Study population

We recruited retrospectively people with musculoskeletal pain from two public physiotherapy departments (i.e., Gaffrée and Guinle University Hospital and Cabo Frio Rehabilitation Centre) and three private physiotherapy departments (i.e., Augusto Motta University Centre, Saúde Clin Physiotherapy Clinic, and Fisiofit Physiotherapy Clinic) in Rio de Janeiro State and Minas Gerais, Brazil between March and September 2019. In public physiotherapy departments, orthopaedists, general practitioners, or other health professionals have often referred people with musculoskeletal pain. People in all private physiotherapy departments reported seeking care for their musculoskeletal condition primarily due to pain.

The study involved people with acute pain (less than three months) and chronic pain (pain duration greater than three months). We defined musculoskeletal pain as pain originating from muscles, ligaments, bones, or joints in a specific body region [24]. We excluded people who had undergone spinal surgery, pregnant women, people in the acute inflammatory phase of rheumatologic diagnoses, people with tumours, those who were illiterate, or those unable to complete the self-reported questionnaires.

### Procedures

The evaluation included the clinical history, physical examination, and cold pressor test performed on the same day for people with musculoskeletal pain. We collected sociodemographic and clinical information using an instrument that included demographic data (age, sex, weight, height, education level, and income) as well as characteristics of musculoskeletal pain (pain intensity and duration). We measured pain intensity using the Numeric Pain Rating Scale, ranging from 0 to 10 (0 represents no pain, and 10 illustrates the worst pain possible). This scale is commonly used in musculoskeletal pain studies and has demonstrated good reproducibility levels [25]. Pain duration was recorded in months, with chronic pain defined as lasting over three months and acute pain lasting less than three months [26]. Neuropathic-like symptoms were measured using the painDETECT questionnaire, with the Brazilian version proving helpful in identifying such symptoms [27]. The cold pressor test assessed the Conditioned Pain Modulation (CPM), which evaluates the descending nociceptive inhibitory system [28, 29]. An examiner supervised the completion of all questionnaires, providing clarification when needed, and the process took approximately 10 min per person with musculoskeletal pain. After completing the questionnaires, musculoskeletal pain patients were referred for CPM evaluation.

### Index method

The PainDETECT questionnaire is a self-administered tool used to assess neuropathic-like symptoms. It comprises four domains with the following components: pain intensity (three questions), pain course pattern (four graphs), areas of pain and presence of radiating pain (body chart drawing), and sensory descriptor items of pain (seven questions). The first domain consists of three questions assessing pain intensity, including the strongest and average pain levels over the past four weeks. The final score is calculated based on a nine-item representation of the last three domains (pain course pattern, radiating pain, and gradation of pain). The second domain (pain course pattern) has answer options of Persistent pain with slight fluctuations = 0, Persistent pain with pain attacks = -1, Pain attacks without pain between them =  + 1, and Pain attacks with pain between them =  + 1. The score for this domain varies between 0 and + 1. The third domain (radiating pain) includes a dichotomous question, "Does your pain radiate to other regions of your body?" with answer options of yes or no, corresponding to scores of + 2 or 0, respectively. The fourth domain (gradation of pain) comprises seven questions, each with six possible answers scored from 0 (never) to 5 (very strongly). The scores given in each domain are summed up to achieve a final score ranging from -1 to 38. The PainDETECT questionnaire is validated for neuropathic pain conditions [30–32] and has also been validated for mixed pain conditions such as rheumatoid arthritis, osteoarthritis, cancer pain, and lumbar spondylolisthesis [33]. The original questionnaire's cut-off points indicate that scores ≤ 12 suggest a neuropathic component is unlikely, scores between 13 and 18 show an unclear neuropathic component, while scores ≥ 19 suggest a probable neuropathic component [33]. For screening purposes, we considered scores ≤ 12 indicative of nociceptive pain, scores between 13 and 18 as unclear, and scores ≥ 19 indicative of neuropathic-like symptoms. The PainDETECT questionnaire was cross-culturally adapted to the Brazilian context [27].

### Reference method

#### The psychophysical measure of the descending nociceptive inhibitory system

We used the cold pressor test as a psychophysical measure to evaluate the descending nociceptive inhibitory system [34] and assess conditioned pain modulation [35]. In this test, the conditioning stimulus was the immersion of the people's hands in a bucket of temperature-controlled cold water (1ºC – 4ºC) for up to one minute. We monitored the water temperature using a thermometer (5130 model, Incoterm). People with musculoskeletal pain were instructed to keep their hands immersed in the water without making muscle contractions or changing positions. They could withdraw their hand from the water when they could no longer tolerate the painful stimulus. We maintained constant room temperature, humidity, lighting, and noise throughout the procedure.

#### Pressure pain threshold

We used a digital pressure algometer (model Force Ten FDX, Wagner Instruments, Greenwich, USA) to measure the pain threshold. We performed the pressure pain threshold assessment before and after one minute of the cold pressor test. The evaluation was conducted on the distal part of the dorsal forearm and tibialis anterior muscle, which had not been immersed in water and were unrelated to people's musculoskeletal complaints. The assessment was done in the same order for all people with musculoskeletal pain. Before the evaluation, we explained the operation of the pressure algometer and how the pressure pain threshold would be measured. We also conducted a familiarisation procedure by applying pressure to the dominant forearm, ensuring the people with musculoskeletal pain understood the test. The force on the algometer was gradually increased (1 kg-force/s) until the primary subject felt a change from pressure to pain. The pressure pain threshold was recorded in kilograms-force (Kgf) when the people with musculoskeletal pain verbally indicated experiencing pain. We classified the efficiency of the conditioned pain modulation based on the following strategy: evidence of impairment of pain modulation in both evaluated sites. Only people with musculoskeletal pain showing impaired conditioned pain modulation in both the anterior tibialis muscle and the distal part of the dorsal forearm were classified as having impaired conditioned pain modulation. Using upper and lower limb sites aimed to avoid including people with peripheral sensitisation, following recommendations for conditioned pain modulation [36]. The efficiency of the conditioned pain modulation was assessed by calculating the difference between the pressure pain threshold values obtained during the cold pressor test (the difference between final and initial values). Negative values indicated an inefficiency of the conditioned pain modulation, while null or positive values were considered a typical response of the conditioned pain modulation.

### Statistical analysis

Demographic and clinical variables of the study population were presented as the mean and standard deviation for continuous variables. Categorical variables were presented as absolute values and frequencies. Independent one-way analysis of variance (ANOVA) was used to test for within and between-group differences (nociceptive pain, unclear, or neuropathic-like symptoms) for the outcome measures with continuous variables (i.e., pressure pain threshold values for the dorsal region of the forearm and anterior tibial of the participants). The diagnostic accuracy of the painDETECT questionnaire (index method) was compared with the psychophysical measure of the descending nociceptive inhibitory system (reference standard). We calculated sensitivity, specificity, likelihood ratio, positive likelihood ratio, negative likelihood ratio, disease prevalence, positive predictive value, negative predictive value, and accuracy with corresponding exact 95% binomial confidence intervals (CIs) for two predefined cutoff points (12 and 19). For diagnostic accuracy tests (i.e., sensitivity, specificity, predictive values, and accuracy), values < 50% were interpreted as low, 50% to 70% as moderate, and > 70% to 100% as high. A significance level of less than 5% (*p* < 0.05) was considered for all analyses. The statistical analysis was performed by Jeffreys's Amazing Statistics Program (JASP), version 0.16.3, and Prism for Macintosh, Version 8 (GraphPad Software Inc., San Diego, CA).

### Sample size calculation

The sample calculation was based on the values obtained from the study by Gervais-Hupé et al. [37]. The authors observed a sensitivity of 61.5% and specificity of 77.6% in identifying impaired conditioned pain modulation using the cutoff point of 12 in the painDETECT questionnaire in people with knee osteoarthritis. The estimate was calculated considering the prevalence of central sensitisation of 21.43% in people with musculoskeletal pain [38], the alpha value of 5%, and the precision of the estimate of 12%. Thus, it was necessary to include 295 people with musculoskeletal pain.

## Results

### Characteristics of the participants

A total of 308 people with musculoskeletal pain were enrolled. Most participants were female (n 20 = 220, 71.4%), had a mean age of 52.2 (± 15.0) years, and had a mean of moderate pain intensity (Table 1). Two-hundred sixty-six (86.3%) participants had chronic pain, and 42 (13.6%) had acute pain. Overall, 43 (13.9%) people with musculoskeletal pain reported a previous diagnosis of fibromyalgia, 48 (15.5%) people with musculoskeletal pain described migraine, 80 (25.9%) people with musculoskeletal pain had anxiety and 73 (23.7%) people with musculoskeletal pain had a prior history of depressive disorder. Low back pain (*n* = 166, 53.8%) was the leading complaint, followed by upper back (*n* = 136, 44.1%), right shoulder (*n* = 131, 42.5%), neck (*n* = 123, 39.9%) and left shoulder (*n* = 116, 37.6%).

**Table 1:** Characteristics of the study people with musculoskeletal pain (*n* = 308)

Characteristics	Values (*n* = 308)
Sex (female), n (%)	220 (71.4%)
Age (years), mean (SD)	52.2 (± 15.0)
Weight (kg), mean (SD)	73.3 (± 16.6)
Height (meters), mean (SD)	1.6 (± 0.1)
Body Mass Index (Kg/m^2^), mean (SD)	27.8 (± 13.1)
Private Health Insurance, yes, n (%)	71 (23.0%)
Physical Activity, yes, n (%)	159 (51.6%)
Pain characteristics	
Pain intensity at the moment, mean (SD)	5.8 (± 2.4)
Strongest pain level in the last 4 weeks, mean (SD)	8.0 (± 2.0)
Pain level on average in the last 4 weeks, mean (SD	6.6 (± 2.2)
Pain duration (months), mean (SD)	66.7 (± 100.7)
Pain intensity, mean (SD)	
Final painDETECT score, mean (SD)	11.9 (± 7.7)
Nociceptive pain (0–12), n (%)	173 (56.1%)
Unclear (13-18), n (%)	69 (22.4%)
Neuropathic-like symptoms (**≥ **19), n (%)	66 (21.4%)
Cold pressor test, impaired, n (%)	60 (19.4%)

Data are presented as mean (SD) for continuous variables and as frequency counts (%) for categorical variables

**Fig. 1:**
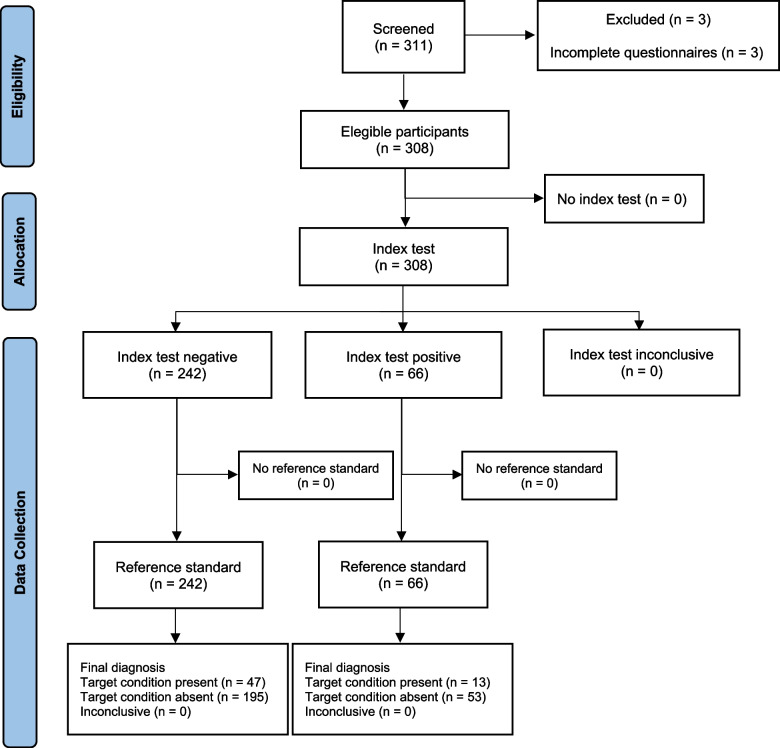
Flowchart of the study

Sixty-six (21.4%) people with musculoskeletal pain had painDETECT questionnaire scores ≥ 19, being 5 (7.5%) classified as acute pain, and 69 (22.4%) people with musculoskeletal pain had painDETECT questionnaire scores between 13–18 points, being 12 (17.3%) classified as acute pain. All people with musculoskeletal pain completed the painDETECT questionnaire and the cold pressor test. Then, there were no missing values for the painDETECT questionnaire and the cold pressor test results. No adverse events were associated with the painDETECT questionnaire and the cold pressor test Fig. 1.

Table 2 presents pressure pain threshold values ​​for people with musculoskeletal pain in the dorsal region of the forearm and anterior tibial. The pressure pain threshold at the anterior tibial before the cold pressor test was reduced in people with unclear classification and neuropathic-like symptoms compared to people with nociceptive pain. The pressure pain threshold at the dorsal forearm pressure after the cold pressor test was reduced in the people classified as unclear and with neuropathic-like symptoms compared to people with nociceptive pain. There is no significant difference in within-group comparison in the dorsal region of the forearm and anterior tibial of the people with musculoskeletal pain.

**Table 2:** Comparison of pain threshold values between people with neuropathic-like symptoms, nociceptive pain, and unclear classification

Characteristics	Nociceptive pain *n* = 173	Unclear *n* = 69	Neuropathic-like symptoms *n* = 66	ANOVA	Comparison Groups	*p*-value
**Baseline**						
Dorsal forearm algometry (kgf)	4.1 (1.5)	3.6 (1.2)	3.6 (1.2)	5.290	Nociceptive *versus* Unclear	.029
					Nociceptive *versus* Neuropathic	.024
Tibialis anterior algometry (kgf)	4.6 (1.7)	4.2 (1.7)	3.5 (1.4)	8.673	Nociceptive *versus* Neuropathic	< .001
**After Cold Pressor Test**						
					Nociceptive *versus* Unclear	.042
Dorsal forearm algometry (kgf)	4.4 (1.6)	3.9 (1.6)	3.6 (1.3)	8.417	Nociceptive *versus* Neuropathic	< .001
Tibialis anterior algometry (kgf)	4.9 (1.9)	4.5 (2.0)	4.0 (1.5)	3.961	Nociceptive *versus* Neuropathic	.003
**Within-group change**						
Dorsal forearm algometry (kgf)	0.3 (1.2)	0.3 (1.1)	-0.0 (1.1)	1.829	-	.162
Tibialis anterior algometry (kgf)	0.3 (1.3)	0.3 (1.1)	0.4 (1.1)	0.221	-	.802

Data are presented as mean (SD) for continuous variables

### Diagnostic accuracy of the painDETECT questionnaire

The cutoff point 12 of the painDETECT questionnaire showed sensitivity, specificity, accuracy below 70%, and a high negative predictive value. The cutoff point 19 of the painDETECT questionnaire showed low sensitivity, high specificity, and high negative predictive value, despite the accuracy below 70% compared to the cold pressor test. Measures of sensitivity, specificity, likelihood ratios, disease prevalence, predictive values, and accuracy regarding the predefined cutoff point of the painDETECT questionnaire for the detection of impairment of the conditioned pain modulation are shown in Table 3.

**Table 3:** Sensitivity, specificity, positive predictive value, negative predictive value, and accuracy are the two predefined cutoff points of the painDETECT questionnaire for detecting of impairment of the conditioned pain modulation

	**painDETECT** **12**	**painDETECT** **19**
Sensitivity %, (95% CI)	46.6% (33.6 – 60.0)	21.6% (12.0 – 34.2)
Specificity %, (95% CI)	43.1% (36.8 – 49.5)	78.6% (73.0 – 83.5)
Positive Likelihood Ratio (95% CI)	0.8 (0.6 – 1.1)	1.0 (0.5 – 1.7)
Negative Likelihood Ratio (95% CI)	1.2 (0.9 – 1.6)	1.0 (0.8 – 1.1)
Impaired CPM Prevalence %, (95% CI)	19.4% (15.2 – 24.3)	19.4% (15.2 – 24.3)
Positive Predictive Value (95% CI)	16.5% (12.9 – 21.0)	19.7 (12.5 – 29.5)
Negative Predictive Value (95% CI)	76.9% (71.7 – 81.5)	80.5% (78.1 – 82.7)
Accuracy (95% CI)	43.8% (38.2 – 49.5)	67.5% (61.9 – 72. 7)

*Abbreviation*: *CPM* Conditioned pain modulation

## Discussion

This study investigated the diagnostic accuracy of the painDETECT questionnaire in identifying the impairment of conditioned pain modulation in people with musculoskeletal pain. Our findings revealed that the painDETECT questionnaire exhibited low sensibility and high specificity for the cutoff point 19 only compared to the cold pressor test. Our data showed high negative predictive values for both cutoffs of the painDETECT questionnaire, which suggests that a negative test can reliably exclude the impairment of the conditioned pain modulation in this population of musculoskeletal pain people. The low prevalence of the impairment of the conditioned pain modulation in the study sample likely increases the negative predictive value. Accordingly, many people with shared values on the painDETECT questionnaire were diagnosed with preserved conditioned pain modulation.

Our findings showed that values below 19 points in the painDETECT questionnaire correctly detect preserved conditioned pain modulation in most people with musculoskeletal pain. Likewise, a previous study considered scores above 18 in the painDETECT questionnaire as CS-related signs and symptoms [39]. The painDETECT questionnaire has been used for the neurobiological confirmation of central sensitisation in people with features of neuropathic pain [40]. However, a definitive cutoff is not a consensus. The cutoff of 12 in the modified painDETECT questionnaire presents a sensitivity of 61.5% and specificity of 77.6% in identifying CS-related signs and symptoms in people with knee osteoarthritis [37]. The exact cutoff has been advised to indicate CS-related signs and symptoms in people with chronic painful knee osteoarthritis [22]. Nonetheless, considering the relatively low sensitivity and specificity measures, the authors did not recommend this cutoff [22]. Similarly, our data suggested that the cutoff of 12 points had insufficient accuracy in identifying the impairment of the conditioned pain modulation in a heterogeneous sample of people with musculoskeletal pain. Moreover, the low values of likelihood ratios for both cutoff points and the low accuracy for cutoff point 12 limit the applicability of the painDETECT questionnaire to identify the impairment of the conditioned pain modulation in people with musculoskeletal pain.

Neuropathic-like symptoms are linked with peripheral and CS-related signs and symptoms. Some genetic variants could be an essential modulator in developing CS-related signs and symptoms in neuropathic pain [41]. Still, CS-related signs and symptoms manifest most in painful conditions with the neuropathic component [42, 43]. Many strategies, such as conditioned pain modulation, could assess clinical features of CS-related signs and symptoms. Conditioned pain modulation is a predictor in developing and treating neuropathic pain [44] but may perform dissimilarly in neuropathic pain conditions. Gagné et al. suggested that the presence of neuropathic pain leads to a decrease in conditioned pain modulation over time [45]. Carpal tunnel syndrome [13] and painful peripheral neuropathy [15] are examples of impairment of conditioned pain modulation. On the other hand, people with painful diabetic neuropathy had a preserved conditioned pain modulation despite pain duration [46]. Hence, the relationship between conditioned pain modulation and neuropathic pain may be a particular feature of this population.

Our study provides new insight for implementation in clinical use and further studies. PainDETECT questionnaire can be used as an initial screening strategy by physiotherapists and other health professionals to screen neuropathic-like symptoms in people with musculoskeletal pain. Similar pain characteristics are present in musculoskeletal pain conditions. Using the painDETECT questionnaire, the physiotherapist can classify the people according to the pain phenotype. Accordingly, the physiotherapist can offer adequate treatment strategies to a given person.

Researchers should use instruments with high accuracy to assess the presence of CS-related signs and symptoms and neuropathic-like symptoms to confirm the present study's findings. Moreover, future studies should concentrate on methods to pragmatically characterise people with impairment of conditioned pain modulation to facilitate the decision-making of physiotherapists. Finally, the diagnostic accuracy of the painDETECT is only one of the considerations when determining a screening tool for musculoskeletal pain. Therefore, additional aspects should be considered.

### Strengths and limitations of the study

We acknowledge the strengths and limitations of the present study. First, this study’s originality verified the diagnostic accuracy of the painDETECT questionnaire to detect impairment of conditioned pain modulation. Second, we used conditioned pain modulation, a reliable measure [47] through a psychophysical test (cold pressor test), to detect the impairment of the conditioned pain modulation using two different anatomical regions to ensure the appropriate classification of the participants. Finally, the large sample size can be considered a strength of this study. The main limitation of the study is that the cold pressor test and the painDETECT questionnaire are not gold-standard for diagnosing the impairment of conditioned pain modulation and neuropathic pain, respectively. Treede suggested that an experiment with secondary hyperalgesia induced by intradermal capsaicin injection is the only documented occurrence of central sensitisation that meets its formal definition [48]. Nevertheless, the cold pressor test is the most common method used [35] and has good to excellent intra-session reliability in healthy and people with chronic pain [49] for conditioned pain modulation assessment. Also, the painDETECT questionnaire can identify neuropathic-like symptoms, but positive neuropathic classification in the painDETECT is insufficient to classify neuropathy [50].

## Conclusion

The painDETECT questionnaire seems valuable for ruling out people with musculoskeletal pain and impairment of conditioned pain modulation.

### Supplementary Information

**Additional file 1. **STARD checklist.

## Data Availability

Not applicable.

## Abbreviations

ANOVA: Analysis of variance
CI: Confidence Interval
CS: Central Sensitisation
CPM: Conditioned Pain Modulation
JASP: Jeffreys's Amazing Statistics Program
Kg: Kilograms
Kgf: Kilograms-force
Kg/m^2^: Kilograms-force per square meter
SD: Standard Deviation
STARD: Standards for Reporting of Diagnostic Accuracy Studies

## References

[1] Bittencourt JV, Bezerra MC, Pina MR, Reis FJJ, de Sá FA, Nogueira LAC (2022). Use of the painDETECT to discriminate musculoskeletal pain phenotypes. Arch Physiother.

[2] Guntel M, Huzmeli ED, Melek I (2021). Patients With Neuropathic Pain Have Poor Sleep Quality. J Nerv Ment Dis.

[3] Verriotis M, Peters J, Sorger C, Walker SM (2021). Phenotyping peripheral neuropathic pain in male and female adolescents: pain descriptors, somatosensory profiles, conditioned pain modulation, and child-parent reported disability. Pain.

[4] Melek LN, Smith JG, Karamat A, Renton T (2019). Comparison of the neuropathic pain symptoms and psychosocial impact of Trigeminal Neuralgia and Painful Post-Traumatic Trigeminal Neuropathy. J Oral Facial Pain Headache.

[5] Cherif F, Zouari HG, Cherif W, Hadded M, Cheour M, Damak R (2020). Depression prevalence in neuropathic pain and its impact on the quality of life. Pain Res Manag.

[6] Freynhagen R, Baron R, Gockel U, Tölle TR (2006). Pain DETECT: a new screening questionnaire to identify neuropathic components in patients with back pain. Curr Med Res Opin.

[7] Freynhagen R, Tölle TR, Gockel U, Baron R (2016). The painDETECT project - Far more than a screening tool on neuropathic pain. Curr Med Res Opin.

[8] Hiyama A, Katoh H, Sakai D (2017). Clinical impact of JOABPEQ mental health scores in patients with low back pain: analysis using the neuropathic pain screening tool painDETECT. J Orthop Sci.

[9] Middlebrook N, Rushton AB, Abichandani D, Kuithan P, Heneghan NR, Falla D (2021). Measures of central sensitization and their measurement properties in musculoskeletal trauma: A systematic review. Eur J Pain.

[10] Mertens MG, Hermans L, Crombez G, Goudman L, Calders P, Van Oosterwijck J (2021). Comparison of five conditioned pain modulation paradigms and influencing personal factors in healthy adults. Eur J Pain.

[11] Bannister K, Dickenson AH (2016). What the brain tells the spinal cord. Pain.

[12] Ossipov MH, Morimura K, Porreca F (2014). Descending pain modulation and chronification of pain. Curr Opin Support Palliat Care.

[13] Arias-Buría JL, Ortega-Santiago R, De-la-Llave-Rincón AI. Understanding central sensitization for advances in management of carpal tunnel syndrome. 2020;9:1–7. 10.12688/f1000research.22570.1 PMC7308881 32595941

[14] Niesters M, Proto PL, Aarts L, Sarton EY, Drewes AM, Dahan A (2014). Tapentadol potentiates descending pain inhibition in chronic pain patients with diabetic polyneuropathy. Br J Anaesth.

[15] Niesters M, Aarts L, Sarton E, Dahan A (2013). Influence of ketamine and morphine on descending pain modulation in chronic pain patients: A randomized placebo-controlled cross-over proof-of-concept study. Br J Anaesth.

[16] Seifert F, Kiefer G, Decol R, Schmelz M, Maihöfner C (2009). Differential endogenous pain modulation in complex-regional pain syndrome. Brain.

[17] Lluch E, Torres R, Nijs J, Van Oosterwijck J (2014). Evidence for central sensitization in patients with osteoarthritis pain: a systematic literature review. Eur J pain.

[18] Sasaki E, Ota S, Chiba D, Kimura Y, Sasaki S, Ando M (2021). Association between central sensitization and increasing prevalence of nocturnal knee pain in the general population with osteoarthritis from the Iwaki Cohort Study. J Pain Res.

[19] Chimenti RL, Frey-Law LA, Sluka KA (2018). A mechanism-based approach to physical therapist management of pain. Phys Ther.

[20] Moreton BJ, Tew V, Das Nair R, Wheeler M, Walsh DA, Lincoln NB (2015). Pain phenotype in patients with knee osteoarthritis: Classification and measurement properties of painDETECT and self-report leeds assessment of neuropathic symptoms and signs scale in a cross-sectional study. Arthritis Care Res.

[21] Gwilym SE, Keltner JR, Warnaby CE, Carr AJ, Chizh B, Chessell I (2009). Psychophysical and functional imaging evidence supporting the presence of central sensitization in a cohort of osteoarthritis patients. Arthritis Care Res.

[22] Hochman JR, Davis AM, Elkayam J, Gagliese L, Hawker GA (2013). Neuropathic pain symptoms on the modified painDETECT correlate with signs of central sensitization in knee osteoarthritis. Osteoarthr Cartil.

[23] Bossuyt PM, Reitsma JB, Bruns DE, Bruns DE, Glasziou PP, Irwig L (2015). STARD 2015: An updated list of essential items for reporting diagnostic accuracy studies1. Radiology.

[24] Murray CCJL, Abraham J, Ali MK, Alvarado M, Atkinson C, Baddour LM, et al. The state of US health, 1990–2010: burden of diseases, injuries, and risk factors. JAMA - J Am Med Assoc. 2013;310(6):591–608. Available from: https://jama.jamanetwork.com/data/Journals/JAMA/927436/joi130037.pdf%5Cnhttp://ovidsp.ovid.com/ovidweb.cgi?T=JS&PAGE=reference&D=emed11&NEWS=N&AN=2013503627%5Cnhttp://ovidsp.ovid.com/ovidweb.cgi?T=JS&PAGE=reference&D=medl&NEWS=N&AN=23842577. 10.1001/jama.2013.13805 PMC5436627 23842577

[25] Hawker GA, Mian S, Kendzerska T, French M (2011). Measures of adult pain: Visual analog scale for pain (vas pain), numeric rating scale for pain (nrs pain), mcgill pain questionnaire (mpq), short-form mcgill pain questionnaire (sf-mpq), chronic pain grade scale (cpgs), short form-36 bodily pain scale (sf. Arthritis Care Res (Hoboken).

[26] Merskey H, Bogduk N. International association for the study of pain. Task force on taxonomy. Classification of chronic pain: descriptions of chronic pain syndromes and definitions of pain terms. 1994.

[27] do Rio JPM, Bittencourt JV, Corrêa LA, Freynhagen R, dos Reis FJJ, de Melo TB, et al. Cross-cultural adaptation of the painDETECT questionnaire into Brazilian Portuguese. Braz J Anesthesiol. 2022;72:44–48. 10.1016/j.bjane.2021.06.013 PMC9373606 34229028

[28] Vaegter HB, Handberg G, Kent P (2017). (345) Brief psychological screening questions can be useful for ruling out psychological conditions in patients with chronic pain. J Pain.

[29] Kent P, Mirkhil S, Keating J, Buchbinder R, Manniche C, Albert HB (2014). The concurrent validity of brief screening questions for anxiety, depression, social isolation, catastrophization, and fear of movement in people with low back pain. Clin J Pain..

[30] Cappelleri JC, Koduru V, Bienen EJ, Sadosky A (2016). Characterizing neuropathic pain profiles: enriching interpretation of painDETECT. Patient Relat Outcome Meas..

[31] Packham TL, Cappelleri JC, Sadosky A, MacDermid JC, Brunner F (2017). Measurement properties of painDETECT: Rasch analysis of responses from community-dwelling adults with neuropathic pain. BMC Neurol.

[32] Abu-Shaheen A, Yousef S, Riaz M, Nofal A, AlFayyad I, Khan S (2018). Testing the validity and reliability of the Arabic version of the painDETECT questionnaire in the assessment of neuropathic pain. PLoS ONE.

[33] Freynhagen R, Tölle TR, Gockel U (2016). The painDETECT project–far more than a screening tool on neuropathic pain. Curr Med Res Opin.

[34] Lewis GN, Heales L, Rice DA, Rome K, McNair PJ (2012). Reliability of the conditioned pain modulation paradigm to assess endogenous inhibitory pain pathways. Pain Res Manag.

[35] Lewis GN, Rice DA, McNair PJ (2012). Conditioned pain modulation in populations with chronic pain: a systematic review and meta-analysis. J pain..

[36] Yarnitsky D, Bouhassira D, Drewes AM, Fillingim RB, Granot M, Hansson P (2015). Recommendations on practice of conditioned pain modulation (CPM) testing. Eur J Pain (United Kingdom).

[37] Gervais-Hupé J, Pollice J, Sadi J, Carlesso LC (2018). Validity of the central sensitization inventory with measures of sensitization in people with knee osteoarthritis. Clin Rheumatol.

[38] Nogueira LAC, Chaves ADO, Wendt ADS, De SRLS, Reis FJJ, De AFG (2016). Central sensitization patients present different characteristics compared with other musculoskeletal patients: A case–control study. Eur J Physiother.

[39] Rifbjerg-Madsen S, Christensen AW, Boesen M, Christensen R, Danneskiold-Samsøe B, Bliddal H (2014). Can the painDETECT Questionnaire score andMRI help predict treatment outcome in rheumatoid arthritis: Protocol for the Frederiksberg hospital’s Rheumatoid Arthritis, pain assessment and Medical Evaluation (FRAME-cohort) study. BMJ Open.

[40] Soni A, Wanigasekera V, Mezue M, Cooper C, Javaid MK, Price AJJ (2019). Central Sensitization in Knee Osteoarthritis: Relating Presurgical Brainstem Neuroimaging and PainDETECT-Based Patient Stratification to Arthroplasty Outcome. Arthritis Rheumatol.

[41] Sachau J, Bruckmueller H, Gierthmühlen J, Magerl W, May D, Binder A (2021). The serotonin receptor 2A (HTR2A) rs6313 variant is associated with higher ongoing pain and signs of central sensitization in neuropathic pain patients. Eur J Pain.

[42] Freynhagen R, Baron R (2009). The evaluation of neuropathic components in low back pain. Curr Pain Headache Rep.

[43] Woolf CJ (2011). Central sensitization: implications for the diagnosis and treatment of pain. Pain.

[44] Granovsky Y (2013). Conditioned pain modulation: a predictor for development and treatment of neuropathic pain. Curr Pain Headache Rep.

[45] Gagné M, Côté I, Boulet M, Jutzeler CR, Kramer JLK, Mercier C (2020). Conditioned pain modulation decreases over time in patients with neuropathic pain following a spinal cord injury. Neurorehabil Neural Repair.

[46] Granovsky Y, Nahman-Averbuch H, Khamaisi M, Granot M. Efficient conditioned pain modulation despite pain persistence in painful diabetic neuropathy. Pain Rep. 2017;2(3):1–7. 10.1097/PR9.0000000000000592 PMC5741298 29392208

[47] Kennedy DL, Kemp HI, Ridout D, Yarnitsky D, Rice ASC (2016). Reliability of conditioned pain modulation: A systematic review. Pain.

[48] Treede RD (2016). Gain control mechanisms in the nociceptive system. Pain.

[49] Nuwailati R, Bobos P, Drangsholt M, Curatolo M (2022). Reliability of conditioned pain modulation in healthy individuals and chronic pain patients: a systematic review and meta-analysis. Scand J Pain.

[50] Hasvik E, Haugen AJ (2019). Call for Caution in Using the Pain DETECT Questionnaire for Patient Stratifi cation Without Additional Clinical Assessments: Comment on the Article by Soni et al. Arthritis Rheumatol.

